# Visualization of Retroviral Gag-Genomic RNA Cellular Interactions Leading to Genome Encapsidation and Viral Assembly: An Overview

**DOI:** 10.3390/v14020324

**Published:** 2022-02-05

**Authors:** Serena Bernacchi

**Affiliations:** Architecture et Réactivité de l’ARN-UPR 9002, IBMC, CNRS, Université de Strasbourg, F-67000 Strasbourg, France; s.bernacchi@ibmc-cnrs.unistra.fr

**Keywords:** retrovirus, Gag precursor, genomic RNA packaging, retroviral assembly, ribonucleoprotein complex, specific interactions, plasma membrane, cellular trafficking, live-cells microscopy, fluorescent labelling

## Abstract

Retroviruses must selectively recognize their unspliced RNA genome (gRNA) among abundant cellular and spliced viral RNAs to assemble into newly formed viral particles. Retroviral gRNA packaging is governed by Gag precursors that also orchestrate all the aspects of viral assembly. Retroviral life cycles, and especially the HIV-1 one, have been previously extensively analyzed by several methods, most of them based on molecular biology and biochemistry approaches. Despite these efforts, the spatio-temporal mechanisms leading to gRNA packaging and viral assembly are only partially understood. Nevertheless, in these last decades, progress in novel bioimaging microscopic approaches (as FFS, FRAP, TIRF, and wide-field microscopy) have allowed for the tracking of retroviral Gag and gRNA in living cells, thus providing important insights at high spatial and temporal resolution of the events regulating the late phases of the retroviral life cycle. Here, the implementation of these recent bioimaging tools based on highly performing strategies to label fluorescent macromolecules is described. This report also summarizes recent gains in the current understanding of the mechanisms employed by retroviral Gag polyproteins to regulate molecular mechanisms enabling gRNA packaging and the formation of retroviral particles, highlighting variations and similarities among the different retroviruses.

## 1. Introduction

Retroviral assembly is a finely tuned process that requires viral and cellular factors to converge at the right time at defined cellular sites to be efficiently achieved. To generate an infectious particle, retroviruses must selectively package two homologous copies of their single stranded gRNA of positive polarity that are non-covalently linked through intermolecular base-pairing. The dimeric gRNA is selected from a much larger pool of cellular and sub genomic viral RNA moieties (for reviews see [1,2,3]), and this specific selection is achieved through the recognition of cis-acting genomic packaging signals (Psi or ψ) by the main structural polyprotein Gag. Retroviral Gag are present in all the members of the *Retroviridae* family (for reviews see [4,5,6,7]), and their expression is considered as sufficient for the in vitro assembly of retroviral like-particles (VLPs). To this aim, Gag polyproteins employ mechanisms involving their structural domains in association with several viral and host factors including lipid membranes, proteins and RNAs. Some determinants for gRNA encapsidation as gRNA dimerization are common to all different types of retroviruses. On the other hand, retroviruses display two major pathways of assembly: B/D-type retroviruses, such as the Mason-Pfizer Monkey Virus (MPMV), assemble immature procapsids at a pericentriolar location, which are then trafficked to the plasma membrane (PM) where budding takes place [8], while C-type retroviruses, such as the lentivirus Human Immunodeficiency Virus (HIV-1) and gammaretroviruses as the Murine Leukemia Virus (MLV), assemble and bud from sites on PM.

Retroviral life cycles, and especially the HIV-1 one, have been previously studied by combining tools derived from biochemistry, molecular biology, genetic and structural biology; however, these methods have only yielded a partly incomplete spatio-temporal view of gRNA packaging and retroviral assembly. However, in the last decades, several bioimaging tools provided important insights gained from the direct visualization of viral processes in live cells. Quantitative bioimaging microscopic approaches based on the fluorescent labelling of the different viral components (e.g., Gag and viral RNA) resulted in considerable gains in our knowledge of molecular mechanisms leading to the formation of retroviral particles. Accordingly, data acquired in the lasts 20 years allowed for the monitoring of the real-time intracellular movements of retroviral Gag-gRNA complexes, and provided new understandings at high spatial resolution of determinants regulating the accumulation of ribonucleoprotein complexes at the budding sites at PM [9,10,11,12,13,14,15,16,17,18,19,20].

HIV-1 is the most well characterized retrovirus, as attested by a huge amount of studies [10,12,14,21,22,23]. Here, the current knowledge based on these recent quantitative microscopic methods of when and where retroviral HIV-1 Gag proteins recruit the gRNA dimer for encapsidation and orchestrate the assembly of the viral particle is summarized. Finally, in this review, variations and similarities between HIV-1 and other retroviruses, as the prototypic simple retroviruses MLV and Rous Sarcoma Virus (RSV) as well as the complex Feline Immunodeficiency Virus (FIV), are also presented.

## 2. Retroviral Gag Precursors

The initial recognition between HIV-1 Gag (Pr55^Gag^) and viral RNA is driven by highly specific interactions between Gag and Psi. Conversely, the achievement of the encapsidation of up to 2400 molecules of Gag [24] around a gRNA dimer to form a viral particle is thought to rely on Gag-gRNA non-specific interactions [25,26,27]. The virion also contains over a hundred Gag-Pol precursors, and therefore the Gag-Pol/Gag stoichiometry is about 1/20 [28]. Shortly after budding, retroviral maturation occurs and the viral protease (PR) separates the precursor into its different domains by a proteolytic cleavage, which renders the virus mature and infectious. Retroviral Gag precursors are composed of independently-folded multifunctional domains that enable interactions with other Gag proteins, and with viral and cellular proteins, membrane lipids, DNA and RNA (NA) (reviewed in [29]). Three domains are found in all Gag proteins: they are matrix (MA) at the N-terminus, capsid (CA) and nucleocapsid (NC). The major function of MA resides in its ability to anchor Gag proteins to the PM where the virions bud. Most retroviral Gag proteins bear an N-terminal myristyl group which is post-translationally added to the amino-terminal glycine residue in MA (for a review see [30]), and which allows hydrophobic interactions with the PM. Zhou and Resh proposed that specific Gag binding to the PM is regulated by a mechanism named “myristyl switch [31], and several other groups analyzed the molecular mechanisms promoting the exposure of the myristyl group for its insertion into the PM [32,33,34,35]. MA interaction with PM can also be regulated by its highly basic region (HBR). This last one enables electrostatic interactions with negatively charged PM-specific acidic phospholipids, such as the phosphatidylinositol-(4,5)-bisphosphate [PI(4,5)P(2)] [36,37]. The HBR-PI(4,5)P2 interaction acts as a trigger of the myristyl switch [38] and modulates Gag chaperone activity [39]. HBR can bind NA, and tRNAs constitute the majority of cellular RNAs bound to Gag in the cytosol [25]. Interestingly, the NC domain seems to facilitate the loading of specific tRNAs onto MA [40]. These HBR-NA interactions interfere with the interactions between MA and acidic lipids, and reduce the myristate exposure to prevent premature association of Gag with cellular membranes, thus temporally regulating Gag localization in the cell [41]. However, it is also possible that tRNA binding and myristate exposure are reciprocally regulated [42]. The myristate exposure was also found to be coupled with Gag multimerization, as the myristyl group is sequestered in the monomer [33]. At the assembly sites where Gag multimerization becomes more extensive, Gag was found to adopt an extended linear conformation which is required to form the Gag lattice and which is promoted by inositol hexakisphosphate (IP6) [43,44]. In turn, this extended linear conformation is thought to promote Gag oligomerization [2,3].

The formation of the retroviral lattice is mediated by Gag-Gag interactions that are mainly driven by CA domains. Interestingly, cryo-electron tomography analysis provided a detailed structural picture of CA domains in immature assembled particles showing that, within the assembled lattices of HIV-1, MPMV, and RSV, the C-terminal domains of CA (CTD) adopt similar quaternary arrangements, while the N-terminal domains (NTD) are packed in a very different manner, thus exhibiting a structural diversity of this domain [45]. Indeed, several studies have shown that the mutations affecting the CTD of CA impair the virion assembly, while the NDT does not seem to be required, at least in the early stages of the process [46,47,48,49]. Accordingly, in HIV-1 two residues within the dimerization interface in the CA-CTD were identified as crucial for Gag-Gag interactions (W184 and M185) [28,29]. Interestingly Gag-RNA interactions and Gag multimerization are linked processes, as HIV-1 Gag interaction with RNA is stabilized by Gag-Gag interactions regulated by the CTD [3,26]. In line with this idea, previous analysis in RSV revealed that the interactions between Gag (Pr76^Gag^) and oligonucleotides of various lengths regulate the precursor association as dimers, and this oligomer intermediate was proposed to trigger further Gag polymerization to complete the viral shell of the immature virions [50].

NC is the putative domain for specific interactions with gRNA leading to genome selection and packaging into the viral particle. In the HIV-1 context, NC actively contributes to Gag multimerization [51], and unpairing the interactions between NC and gRNA was observed to induce aberrant DNA-containing viruses [52,53]. However, retroviral NC domains were observed to exhibit different implications for gRNA packaging as, for example, in deltaretrovirus (as Human T-Cell leukemia virus type 1, HTLV-1, or Bovine Leukemia Virus, BLV) compared to the NC domain of HIV-1 Gag. Therefore, in deltaretrovirus, MA assists NC in genome encapsidation [54], and conversely, the non-myristoylated Gag of the alpharetrovirus RSV makes use of both NC and MA domains to increase the electrostatic interactions with acidic lipids at the PM [55]. These considerations suggest that Gag domains are multivalent, and retroviral precursors adapt their functions efficiently to the different contexts.

**Figure 1:**
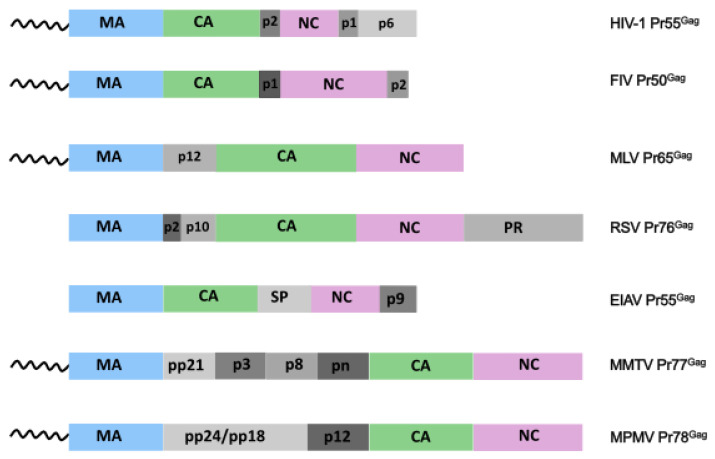
Retroviral Gag precursors. Three domains are found in all the retroviral Gag proteins: matrix (MA, light blue) at the N-terminus, capsid (CA, green) and nucleocapsid (NC, pink). In addition to those main domains, retroviral Gag also possess other small domains (various shades of grey) located at various positions which display different activities.

In addition to those main three domains, other domains located at different positions in retroviral Gag display different functions (Figure 1). The non-canonical p8 domain in Murine Mammalian Tumor Virus (MMTV) Gag (Pr77^Gag^) and the p2 domain in FIV Gag (Pr50^Gag^) play a role in Gag-mediated assembly and particle production [56,57], while the p9 domain in Equine Infectious Anemia Virus (EIAV) Gag (Pr55^Gag^), the p2 domain in RSV Gag (Pr76^Gag^), the p6 domain in HIV-1 Gag (Pr55^Gag^), the pp24/pp18 domain in MPMV Gag (Pr78^Gag^) [58,59], and the pp21 domain in MTMV Gag contain common or alternative conserved motifs termed late domains (or L-domains), that specifically recruit the Endosomal Sorting Complex Required for Transport (ESCRT) machinery at viral budding sites to regulate viral budding of nascent virions at PM (Figure 1, for a review see [60]). Additional retroviral domains exhibit a structural role in viral assembly or in Gag multimerization, as, for instance, the p10 domain in RSV Gag [45], or the segment p2, located between NC and CA in HIV-1 Gag [61,62] as mutations in this domain modulate packaging of spliced viral RNAs [63,64]. Finally Gag domains can also exhibit regulatory functions as the p12 domain in MLV Gag (Pr65^Gag^) that, in its mature form, tethers the pre-integration complex (PIC) to host chromatin for integration [65], the PR domain in RSV Gag that displays an enzymatic protease activity, the p8 domain in MMTV Gag which is mono-ubiquitinated [30], and the p6 domain in HIV-1 Gag that was also observed to affect Gag binding to short oligoribonucleotides [66], and to regulate Gag binding specificity to gRNA fragments [67].

**Figure 2:**
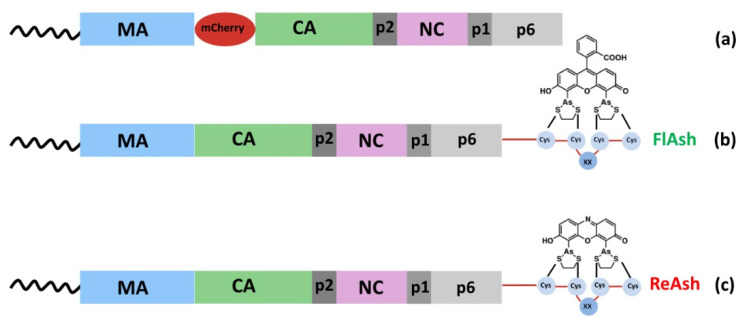
Different labelling strategies allow the detection of Gag precursors in cells. (**a**) Gag fusion to FPs such as m-Cherry was, for example, used to analyze the trafficking of HIV-1 Gag-gRNA complexes to the PM where the assembly occurs [21]. Membrane-permeable biarsenical compounds named (**b**) FlAsH, whose structure is based around a fluorescein core, and (**c**) ReAsH, which is a derivative of resorufin, were also used to observe HIV-1 Gag dynamics [20]. In this case, Gag labelling was achieved by adding a linear tetraCys motif (TC-tag), where XX is usually Pro-Gly [74]. Importantly, this labelling is highly specific, as Gag becomes fluorescent only when the biarsenical compounds bind the target.

Retroviral Gag fusion to fluorescent proteins (FPs) was widely used to analyze the cellular interactions leading to the retroviral assembly (Figure 2). FPs display a vast color palette even though the most commonly used are the Green Fluorescent Protein (GFP) and m-Cherry [21] (Figure 2a). This labelling strategy allows the attachment of the label at a chosen position, and the location of the fusion FP into retroviral Gag has however to be carefully chosen to induce minimal perturbations on viral functions. In the HIV-1 context, the insertion of a fluorescent fusion protein at the C-terminus of Gag is incompatible with GagPol frameshifting, and the insertion at the N-terminus would affect the interaction with the PM. Therefore, the less perturbative site for FPs was found to be between the MA and CA domains, and FP was flanked by two functional protease cleavage sites to allow Gag processing into native-sized polypeptides [16,68]. Notably, this approach was compatible with the assembly and the maturation of viral particles, even if, in absence of helper unlabeled HIV-1 Gag, viral infectivity was reduced [68], Gag-GFP displayed aberrant localization [16], and virus-like particles exhibited abnormal densities in electron microscopy examinations [69,70]. Within the fusion proteins pool, photoconvertible fluorescent proteins constitute a large group of proteins that, when they undergo a light-dependent irreversible chemical conversion, generate an irreversible structural change with the consequence of switching their emission color from green to red (reviewed in [71]). These photoactivatable derivatives were thus particularly suitable for pulse-chase analyses (reviewed in [72,73], and see Section 8). In cell imaging assays, specific Gag binding to membrane-permeable biarsenical compounds named FlAsH and ReAsH (also corresponding to a Förster Resonance Energy Transfer, FRET, couple) were also used to observe HIV-1 Gag dynamics in HeLa and Jurkat T cells [20] (Figure 2b,c). In this case, Gag labelling is achieved by adding a small tetra cysteine-tag (TC-tag), which is made up of 6–12 amino acids and can be specifically stained by biarsenical dyes.

However, it is important to note that protein labelling with fluorescent tags can produce a number of caveats that have to be considered. Indeed, fluorescence intensity might not always reflect the number of chromophores, and thus precisely quantifying the molecules present at a particular location in cells can be challenging. Control experiments are therefore necessary to demonstrate the authenticity of the supposed interactions occurring between labelled factors, and quantitative analysis of particle assembly requires very careful interpretation.

## 3. Gag Oligomerization

Biochemical assays and electron microscopy studies previously indicated the presence of HIV-1 Gag cytoplasmic oligomers [75,76], even though the characterization of their stoichiometry had been rather controversial. In addition, epifluorescence microscopy combined with FRAP (fluorescence recovery after photobleaching) displayed the presence of highly mobile cytoplasmic HIV-1 Gag, likely corresponding to monomers or low-order multimers [26,77]. Conversely, fluorescent fluctuations spectroscopy (FFS) techniques pointed out low-diffusing Gag species (D = 0.014 ± 0.002 µm^2^/s) [14], and both populations were found to nucleate and grow at the assembly sites [11,12,14]. Quantitative-FRET microscopy analysis at PM showed that the disruption of the HIV-1 CA-CTD strongly reduced Gag multimerization, while mutations in NC displayed less-severe defects, thus conferring to these two domains an order of importance in Gag oligomerization [78]. Moreover, truncated Gag proteins, where either the complete NC domain or the NC basic residues were deleted, displayed high cytoplasmic mobility (D = 6.0 ± 1.2 µm^2^/s), which is consistent with the resulting impaired interactions with gRNA and the affected production of viral particles [14]. Therefore, even though RNA does not seem to be mandatory for Gag oligomerization per se, RNA displays a structural scaffolding role that facilitates Gag multimerization through NC, which is in line with several in vitro findings [50,79,80,81,82]. Interestingly, CA and p2 domains were found to be involved in gRNA packaging [83,84], and thus, in turn, HIV-1 Gag multimerization might play a role in the interaction with gRNA in the cytoplasm [25] or at the PM [85]. Conversely, advanced FFS methods showed that an HIV-1 Gag protein defective in membrane binding (Gag G2A) displays in the cytosol similar RNA biding properties and a similar oligomeric state compared to the native form of Gag [14,21,86].

By combining two-photon FRET and FFS, Larson et al. [70] observed RSV Gag multimers in the cytoplasm and also at the PM. Finally, the cytosolic diffusion of monomeric HIV-1 Gag labelled with m-Cherry or Venus fluorescent proteins (D = 2.8 ± 0.5 µm^2^/s) [14], the diffusion of GFP-tagged RSV Gag proteins diffusing in chicken fibroblast cells (D = 3.2 ± 0.6 µm^2^/s) [17], and the diffusion of EYFP-tagged HTLV-1 Gag (D = ∼1–3 µm^2^/s) [86] were found to be similar.

## 4. Retroviral Packaging Signals and gRNA Dimerization

Packaging signals are commonly located at the 5′ end of retroviral gRNAs. They are formed by several stem-loops including the dimerization initiation site (DIS) that mediates base-pairing between two copies of gRNA, and display stretches of unpaired purines that might be potential binding sites for NC [87,88,89]. Moreover, genomic dimerization is thought to be a general prerequisite for retroviral gRNA packaging and, accordingly, aberrant gRNA dimerization was found to impair viral assembly (reviewed in [90,91]). HIV-1 viral spliced RNAs were found to compete with gRNA for packaging, even though they are packaged into viral particles to a lesser extent [90]. The gRNA region spanning nucleotides 227–337 were identified as the core Gag binding domain for specific gRNA packaging. However, these highly specific Gag-viral RNA interactions are regulated by RNA folding, and structural analyses showed base-pairing between the CU-rich regions upstream of SL1 (which is present in both unspliced and spliced viral RNAs) and nucleotides around the AUG codon (which are located downstream of the major splice donor site). These findings thus pinpoint not only how Gag recognizes its primary binding site on the viral RNA, but also how specific Gag binding is negatively regulated in spliced viral RNAs [91].

The Psi region in MLV is formed by four highly structured stem-loop structures displaying different roles: the first two promote gRNAs dimerization, while the last ones lead to non-canonical loop-loop interactions that stabilize the duplex, and form the core encapsidation signal [92,93,94,95]. In RSV, although a bipartite DIS was identified by 2′-hydroxyl acylation analyzed by primer extension (SHAPE) assays, mutations of the identified DIS would not fully impair viral replication, suggesting that this process involves additional RNAs interactions [96]. HIV-1 Psi is constituted of four RNA stem-loops, SL1 to SL4 [97,98], and the HIV-1 dimer is thought to be initiated via the kissing-loop interactions involving conserved palindromic sequences located in the SL1 apical loop [99,100,101,102,103,104]. Other genomic regions such as the Trans-Activation Response element (TAR), the Primer Binding Site (PBS) and the unique sequence in the 5′ site (U5) in the 5′ UTR or sequences within the ORF of Gag were observed to contribute to gRNA dimerization even though they do not participate directly to intermolecular base-pairing (for a review see [4]). Interestingly, a similar feature was recently observed by the group of Rizvi who showed that MMTV PBS actively participate to gRNA packaging [105]. In vitro analysis indicated that the HIV-1 kissing-loop dimer can be refolded into a more stable duplex form [106], and this refolding process is thought to occur through a cruciform intermediate [107], and to be chaperoned by the viral NC protein [108,109,110]. More recently, NMR studies detected a U5: AUG interaction in which U5 bases pair with the *gag* start codon (AUG), thus promoting gRNA dimerization as well as NC protein binding. These findings interestingly supported the idea that dimerization and NC binding are regulated by a common RNA structural switch [111]. This same U5:AUG interaction was found to also promote gRNA dimerization of HIV-2 and of two divergent strains of Simian Immunodeficiency Virus (SIV) [112]. Indeed, long-range interactions were commonly found to regulate retroviral genome dimerization and packaging. As such, the team of Rizvi showed via SHAPE analysis that highly structured stem-loops in MPMV Psi are held together by this conserved long-range interaction between U5 and *gag* complementary sequences [113].

## 5. Visualization of gRNA Dimer in Cell

Recently, fluorescent RNA labelling techniques have been largely used to monitor HIV-1 RNA in the living cell [12,14,15,23,114]. One of the most widely used strategies to visualize RNA in cells consists in introducing stem-loops that are recognized with high affinity and specificity by the bacteriophage MS2 coat protein which is tagged with a fluorescent protein (e.g., GFP). Multiple copies of this stem-loop motif (usually *n* = 24) are thus inserted into the target RNA, which is then expressed in cells in parallel of the nuclear MS2–GFP fusion protein. Thus, RNAs carrying the MS2 stem–loops bind MS2–GFP fusion proteins in the nucleus, and this fluorescent complex then moves to the cytoplasm, where it can be visualized. In an attempt to visualize HIV-1 genomes in cells, several groups inserted MS2 stem-loops within the *gag* intron in order to label only unspliced viral mRNA [10,11,21,114,115,116]. Importantly, these modified viral genomes are efficiently incorporated into virions and are compatible with the production of infectious virions.

The group of Hu also engineered HIV-1 genomes containing binding sites for BglG, an antitermination protein in the *E. Coli* bgl operon to obtain, together with the bacteriophage MS2 coat protein labelling, simultaneous double RNA labelling with two distinct fluorescent proteins. This strategy provided evidence that virions can contain RNAs derived from different parental viruses, and this occurs at ratios expected from a random distribution [117]. In addition, this team used similar labelling to analyze the cytosolic diffusion of HIV-1 RNA [22]. The transport of cellular mRNA in the cytoplasm is indeed a complex process, and mRNAs can be transported either by passive diffusion, or by directional movement that is reminiscent of an active transport along the cytoskeleton likely depending on the microtubule network (reviewed in [118,119]). Chen et al. [22] thus measured by single-molecule tracking the cytosolic diffusion coefficient of Bgl-YFP–labeled HIV-1 RNA in HeLa cells (D = 0.07 − 0.3 µm^2^/s), and observed that gRNA mobility is non-directional, but random and indicative of diffusive movements that do not require an intact cytoskeletal structure. These findings are consistent with total internal reflection fluorescence (TIRF) analysis on HIV-1 RNA that showed viral RNA as being highly mobile, thus suggesting that RNA would not be actively transported through the cytoplasm [10]. On the other hand, in the presence of a sufficient amount of Gag for virion assembly, data from the team of Hu showed that the cytoplasmic mobility of HIV-1 RNA would be similar to the mobility observed for gRNAs alone. In an apparent contrast, several other studies observed that gRNA reaches the PM in association with low-order multimers of Gag precursor [11,25,26]. However, these data are not necessary incompatible with each other since, considering the slower diffusion of the gRNA compared to Gag (see session 7), low-order cytolytic oligomers of Gag co-trafficking in association with gRNA could indeed have a minimal impact on viral RNA mobility [14,26].

A hallmark of retroviruses is that gRNAs are packaged as dimers, however at what point following RNA synthesis, or where dimerization occurs is still rather unclear. In vitro assays also showed that HIV-1 RNA fragments can heterodimerize with spliced viral RNAs only when the two RNAs are synthesized simultaneously [120]. These findings suggested that HIV-1 RNA dimerization could occur in the nucleus during transcription, although considering more recent results, this possibility seems unlikely, at least for HIV-1. Indeed, Mougel used a multicolor HIV-1 RNA labelling strategy coupled to single-molecule microscopy technologies to uncover dimerization mechanisms in the three-dimensionality of the cell [23]. This extensive analysis combined several fluorescence techniques, including fluorescence in situ hybridization (FISH), TIRF, 3D-super-resolution structured illumination microscopy, and FFS methods, as fluorescence cross-correlation spectroscopy (FCCS) aimed at monitoring the spatial RNA-RNA association in living cells and the dynamics of these complexes. To detect HIV-1 unspliced gRNA molecules in two colors, the 5′-end of the *lacZ* gene or the 24 repeats of the bacteriophage MS2 stem-loop were introduced within the *pol* gene, and these findings indicated that the dimerization of HIV-1 genome likely initiates in the cytosol [23]. In line with this idea, Moore et al. showed that HIV-1 RNA molecules destined for co-packaging into the same virion select each other mostly within the cytoplasm subsequently to nuclear exit [121]. On the other hand, both the teams of Mougel and Hu observed that the frequency of dimers is enriched near the PM, and the enrichment would last ∼30 min [11,13,15]. These findings could be compatible with an alternative model where HIV-1 RNA dimerization would occur at the PM [12]. However, of note, the highly dynamic cytosol, compared to the more stable environment of PM, renders these estimations very complex. Moreover, in virions dimers, frequencies were increased by a factor of four to five. Therefore, overall, these considerations seem to indicate that HIV-1 RNA dimers would be initially formed in the cytosol to then be stabilized at viral assembly sites.

Conversely, for other retroviruses such as MLV and RSV, gRNA dimerization was proven to occur in the nucleus. Indeed, MLV-spliced viral RNAs were observed to efficiently heterodimerize with the gRNA, and this suggested that MLV RNA dimerization would be coupled to transcription and splicing processes [122,123]. In addition, RSV RNA dimers were recently visualized in cultured quail fibroblasts using single molecule RNA imaging approaches. Subcellular localization analysis revealed that heterodimers are present in the nucleus, in the cytoplasm, and at the PM, indicating that genome dimers can form in the nucleus [124]. Interestingly, mutations of RNA elements promoting gRNA dimerization affect the intracellular trafficking of the viral genome, and result in an aberrant accumulation of gRNA either in the nucleus or in the cytoplasm [125,126].

Another relevant question is whether gRNA dimerization is initiated before or upon gRNA recognition by the Gag precursor. The analysis by Mougel and co-workers indicated that the NC domain in Gag would lead to the recruitment of gRNA dimers to subsequently traffic them to the assembly sites at PM [23]. Previous in vitro data showed that SL1 is the crucial element for the specific interaction with Gag, and gRNA dimerization would positively influence Gag binding, since an RNA fragment carrying mutations in the DIS palindromic sequence was fixed by the protein with a low affinity, compared to an RNA fragment corresponding to the native sequence [27,127]. It is thus possible that Gag selects a preformed dimeric gRNA from the bulk of the cellular and viral RNAs, and the interaction between the NC domain and the gRNA promotes dimer stabilization. This idea is also in agreement with several previous observations reporting HIV-1 Gag/NC chaperone activity on gRNA dimerization (reviewed in [128,129,130]), and the notion that gRNA dimers are preformed prior to encapsidation [12,23,121,131,132].

## 6. Where Is the gRNA Recruited?

The driving force for the encapsidation of gRNA mainly resides in Gag-gRNA interactions, and the specific selection of retroviral gRNA from a cytoplasmic pool of RNAs is regulated by specific RNA conformations which are supposed to expose the RNA high affinity binding sites to the NC domain within the Gag protein [27,91,127]. Some basic questions on retroviral mechanisms leading to gRNA packaging are how, when, and where the genome is recruited for packaging. In the HIV-1 context, two possibilities were explored, and accordingly Gag could recruit the gRNA in the cytoplasm, and in this case the ribonucleoprotein complex would traffic to the PM [21], or alternatively, RNA and Gag could independently reach the PM and the capture of the genome would occur at those sites [12]. Hu and coworkers demonstrated that the host cell machinery driving the transport of HIV-1 RNA out of the nucleus influences RNAs packaging into virions, as RNAs transported using the CRM-1 pathway do not co-package efficiently with RNAs transported by the NXF1 pathway, although Gag can package RNAs from both the two export pathways [121]. Moreover, in the cytoplasm, Chen et al. observed that both translating and non-translating viral RNAs exhibit dynamic movement and can reach the PM, even though Gag would selectively package non-translating RNA [133]. This single-molecule tracking analysis thus supported a model in which individual RNA molecules carry out only one function at a time.

Poeschla et al. used MS2 phage coat protein labelling to track spatial dynamics of primate and non-primate lentiviral genomic RNAs in live cells [114]. Indirect immunofluorescence and live-cell imaging thus revealed that both the lentiviral HIV-1 and FIV gRNAs traffic into the cytoplasm, and this process is Rev-dependent. Likewise, both lentiviral gRNAs were seen at the PM if and only if Gag was present and Psi was intact. However, FIV Gag and gRNA accumulated at the nuclear envelope contrary to HIV-1 Gag, and this marked the main difference between these two lentiviruses. Overall, this imaging analysis suggested that FIV Gag-genomic RNA interactions could initiate at the nuclear pore, and accordingly Gag would escort the genome during its entire cytoplasmic journey to the assembly sites at PM. On the other hand, observations from the group of Bieniasz proposed a model in which a small number of HIV-1 Gag molecules select viral RNAs in the cytoplasm, and once that the Gag-gRNA complex is anchored to the PM, further accumulation of Gag would lead to the subsequent assembly of the complete virion [10], a view in a good agreement with findings of other groups [21,23]. Therefore, Gag-membrane binding, Gag-RNA and Gag-Gag interactions provide similar scaffolding functions in the achievement of the viral particle assembly, and in line with this idea Carlson et al., using artificial membranes, observed by confocal microscopy that the formation of the Gag lattice and the packaging gRNA into new virus particles are linked processes [85].

**Figure 3:**
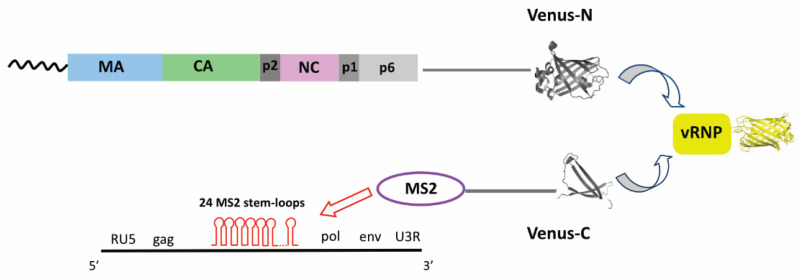
In BiFC assays, the two halves of Venus fluorescent protein are fused to two interacting proteins of interest, as for example Gag and MS2 proteins [147]. One of the most widely used strategies to monitor gRNA trafficking in cells consists in the insertion of 24 stem-loop sequences in the target RNA which are then recognized with high affinity and specificity by the bacteriophage MS2 coat protein. While the two halves of Venus protein are non-fluorescent, the interaction of the partners tethers the fused fluorescent fragments in proximity, which facilitates the restoration of Venus fluorescence. BiFC is a reliable and sensitive approach that also revealed molecular interactions in other viral contexts, such as the Herpes virus [148].

Several retroviral Gag proteins (for a review see [134]) as RSV Gag [135,136,137], MLV Gag [138], Foamy viruses Gag [139], MMTV Gag [140] and MPMV Gag [8] were found in the nucleus, and they are thought to interact with gRNA in this more confined space. In MLV the nuclear export of viral genome is mediated by Gag and occurs on endosomal vesicles [125]. In this retroviral context, Psi stem-loops are supposed to be involved in genome nuclear export and in endosomal transport, suggesting that RNA dimerization is linked to vesicular transport, which is consistent with the proposed requirement for Gag binding [125]. Furthermore, simple retroviruses lack accessory proteins (as for example the HIV-1 Rev that mediates the export of the unspliced gRNA from the nucleus [141]), and therefore, in RSV, Gag displays two nuclear localization signals (NLS) (one involving the importin-α/β pathway, and the other one the transportin SR and the importin-11 [142]) that allow the nuclear export of the unspliced genome. RSV Gag nuclear shuttling was extensively studied (for a review see [143]), and several studies demonstrated that Gag-gRNA interactions promote RSV Gag multimerization [50,80,136,137,144]. In turn, RSV Gag oligomerization produces a conformational change in Gag leading to the exposition of its nuclear export signal (NES), which can then interact with the nuclear export receptor CRM1. This leads the Gag-gRNA complex to cross the nuclear envelope and to be trafficked to the PM for packaging [136,137,145,146]. More recently, the group of Parent visualized Gag-gRNA interactions in the nuclei of infected QT6 quail fibroblast cells [147] by biomolecular fluorescence complementation (BiFC) assays where the two non-fluorescent halves of Venus protein were fused to MS2 and to Gag (Figure 3).

This nuclear interaction, however, leaves many unanswered questions including the possible role of RSV Gag in splicing, in genome expression, or also in chromatin modifications. Interestingly, Parent et al. recently visualized HIV-1 Gag and unspliced viral RNA in discrete foci of Hela cells. At those sites, Gag was found to accumulate at stalled transcription sites. Also, in CD4+ T infected cells treated to stimulate NF-κB mediated transcription, HIV-1 Gag was found to localize at transcriptional burst sites. Altogether these findings open the possibility that also HIV-1 Gag can be found in the nuclei bound to unspliced viral transcripts [149]. Also, HIV-1 Gag was observed to contain an NLS and an NES within the MA, and mutation of the NES would interfere with gRNA packaging [150], but this result has not been further explored. Finally, previous controversial results on the presence of HIV-1 Gag-gRNA complexes outside of the nuclear envelope [114,151] indicate that whether HIV Gag gRNA packaging involves nuclear events has not been fully resolved yet. Therefore, even if it is possible that that formation of HIV-1 vRNP between Gag and gRNA can occur in both the nucleus and the cytoplasm, further studies will be necessary to definitively elucidate this point. One of the limiting factors in these observations resides in the detection limit of fluorescent microscopy, which could be not sensitive enough to visualize small nuclear Gag foci. In future studies, the use of super-resolution imaging techniques in living cells would then be crucial to improve the visualization of vRNPs biogenesis and their trafficking to the PM.

## 7. How Retroviral gRNA-Gag Complexes Are Trafficked to the PM?

A number of fluorescence approaches based on microscopy has been used to investigate the dynamics of protein-protein or protein-RNA complexes in cells during viral assembly (reviewed in [152]). As such, FRET is a suitable method to analyze the interactions occurring between the two chromophores in the range of 2–10 nm (Table 1). Interestingly, the detection of FRET can be monitored through the decrease of the donor fluorescence lifetime in the presence of an acceptor molecule as in FLIM (fluorescence lifetime imaging microscopy, Table 1). This method, together with FFS methods, such as Raster Image Correlation Spectroscopy (RICS) [21], which detects temporal signal fluctuations of the molecules diffusing within a raster scanned image [153], provided information on the intracellular mobility of HIV-1 Gag-gRNA complexes and their accumulation at the PM [21]. It was thus observed that the deletion of both ZF motifs within the NC completely abolished intracytoplasmic Gag-gRNA interactions, while the deletion of either the first or the second ZF delayed the delivery of gRNA to the PM without completely impairing the Gag-gRNA interactions in the cytoplasm. These findings showed that ZFs display redundant roles, even though ZF2 played a more prominent role than ZF1 in the accumulation of the ribonucleoprotein complexes at the PM [21].

Retroviruses have evolved distinct strategies for assembly, and thus RSV and EIAV assembly occur at the PM although their Gag are not myristylated, while MPMV and MMTV Gag proteins are myristylated but the assembly of these retroviruses takes place in the cytoplasm [154,155]. Some retroviruses, such as MLV or HTLV-1 [125,156,157,158] hijack the endosomal machinery and initiate the assembly on endosomal membranes, and subsequently those pre-budding viral complexes are routed to the PM within late endosomes in a microtubule-dependent fashion. However, it is not possible to exclude that Gag-gRNA complexes detected by electron microscopy and immunolabelling could correspond to assembled virions that have been endocytosed [156]. Indeed, after completion of assembly at the PM, MLV viral particles were found to be internalized into CD63-positive highly dynamic endosomes whose mobility is similar of microtubule-based transport [10,11,114]. Even though similar patterns had been previously proposed also for HIV-1 in macrophages, this model was then challenged by several studies (reviewed in [159]). Indeed, electron-microscopy analysis on HIV-1 infected macrophages demonstrated that invaginations of PM were in this case misinterpreted with virus-containing compartments [160]. Similarly, immunofluorescence approaches or subcellular fractionation coupled to pulse-chase labelling showed that HIV-1 Gag is targeted to the PM early after its expression, and inhibitors that abolish late endosome motility did not impair the particle release [9]. Overall, these data as well as recent imaging experiments leading to the visualization of retroviral assembly in real time using spinning disc confocal microscopy or TIRF (see session 8), clarify that HIV-1 assembly occurs at the PM [10,11,13,159].

## 8. Molecular Mechanisms Occurring at the PM

One of the most important challenges to better understand retroviral assembly is to obtain a clear picture of the spatial and temporal organization of the viral and cellular components interacting at the assembly sites. Indeed, opting for an optimal imaging modality to analyse protein-RNA interactions is not simple, and methods providing good spatial resolution are usually less performant in temporal resolution, which is in turn an essential feature for investigating rapid time scales of viral processes. In the past, in addition to subcellular fractionation, and immunofluorescence, electron microscopy (EM) showed that HIV-1 assembly takes place at the PM [76] (Table 1). However, even if this method can resolve finer spatial details compared to the classical light microscopy, cells are fixed, and temporal resolution is completely lost. For this reason, EM was coupled to other methods, such as atomic force microscopy (AFM) which previously allowed to monitor retroviral budding events [161]. This technique is indeed suitable to measure the distortion of the membrane, although with a technically limited temporal resolution of several minutes per frame (Table 1). Temporal information on Gag-gRNA interactions at the PM was also obtained by using photoconvertible fluorescent proteins Eos via an RNA-binding protein that specifically recognizes stem-loops engineered into the HIV-1 viral genome to distinguish between existing and newly arriving viral RNAs at the PM [15]. Accordingly, photoconverted Eos proteins allowed the detection of RNAs at the PM by red signals, while the RNAs that reached the PM later were unmodified and carried green signals. Sardo et al. could thus observe that in the absence of HIV-1 Gag, most of the gRNAs remain transiently at the PM, while in the presence of Gag, the permanence of gRNA at the assembly sites was significantly increased as gRNAs could be still detected after 30 min [15]. In agreement with these findings, Jouvenet et al. observed highly dynamic HIV-1 RNA molecules in the cytoplasm that move rapidly in the proximity of the PM, thus remaining visible in the observation field for no longer than a few seconds. On the other hand, when Gag is co-expressed, a fraction of the RNA molecules is found to dock at the membrane, displaying slow and lateral drift (∼ 0.01 μm/s) [10]. Therefore, the duration of HIV-1 RNA retention at the PM depends on Gag expression, indicating that at the assembly sites gRNA is associated to Gag.

The photoconvertible proteins Eos were also used by Jouvenet et al. to monitor Gag recruitment at the assembly sites. These findings demonstrated that Gag proteins reach the assembly sites mainly from the cytosol, rather than from lateral diffusion on membranes [13]. The assembly of individual virions was also observed in real-time. As such, the gradual recruitment of Gag fused GFP at budding sites was observable as the fluorescence intensity initially followed a saturating exponential, while the end of this phase was marked by a plateau of the fluorescence intensity [11]. As the signal produced by Gag increased, the lateral movements of gRNA molecules were observed to decrease, indicating that Gag accumulation promotes gRNA anchoring to the PM [159]. At those sites remarkably about 75% of the assembly events occur on HIV-1 Gag-gRNA complexes, which is in line with the notion that viral RNA plays a scaffolding role at the assembly sites [10]. Of note, the recruitment of the viral RNA is reversible (whenever the assembly of Gag is disrupted), even though the time observed for viral RNA dissociation from a multimerization defective Gag is greater that the time necessary for the accumulation of Gag at the assembly sites [10].

However, during the assembly process, Gag proteins are constituted by a mixed population of molecules at potentially different stages of assembly, and thus it can be somehow difficult to determine whether a fluorescent signal is produced by assembling Gag proteins or by virions. Moreover, the situation can be also complicated by the fact that retroviral assembly is a rare event that emits a fluorescent signal in a large background. Therefore, the combination of different methods is necessary to obtain a more precise picture of the assembly events. As such, FRAP is a suitable method to analyze cellular mobility of HIV-1 Gag and provides accurate information on immobile fractions [77] (Table 1). Indeed, this method allows the distinguishing between particles that continuously recruit Gag molecules (which displayed increasing fluorescence during the prebleach period, and that recover a signal after bleaching), from particles for which the recruitment was already completed (whose intensity was found to be high and stable during the prebleach period, and that do not recover after bleaching) [11]. Of note, Gag cannot be directly detected in its first association with PM, and on average, ∼ 4.5 min from the appearance of the viral RNA at the PM, are necessary to start observing the signal of Gag proteins [11].

One of the most vastly used methods to analyze the genesis of the individual virions at PM was TIRF [10,11,13,159]. This method indeed displays a sufficient signal-to- noise ratio to allow the quantification of the individual assembling particles, since the illuminated volume corresponds to a shallow region between the glass and the cell, and fluorophores in proximity (≈<70–100 nm) of the coverslip are then excited (Table 1). However, TIRF is also sensitive to the axial position of the fluorescent molecules, and fluctuations of the fluorescence intensity due to movement of the PM could cause difficulties in the measure of the assembly complexes. For this reason, TIRF microscopy was complemented with wide-field observation [13,162], and it was thus observed that the assembly of HIV-1 Gag shell takes ∼200 s [13]. Altogether, these observations are consistent with a model where a small number of Gag molecules (i.e., below the detection limit) interacts with gRNA that is thus immobilized at the PM during the early events of particle assembly. These vRNP complexes therefore constitute the nucleation sites for the recruitment of further Gag molecules [10]. At this stage, Gag conformational changes convert the initial small oligomers into assembly-competent ones, and retroviral particle assembly is achieved through a set of larger intermediates. The time-lapse between the first recruitment of gRNA to the PM (by a small number of HIV-1 Gag) and the completion of the viral particle at the assembly sites (which corresponds to the termination of Gag accumulation) was estimated between to be between six and 27 min by Jouvenet et al. [159]. This estimation is in agreement with other observations indicating that signals reporting HIV-1 Gag accumulation at the PM take five to 30 min to reach their maximal intensity [13,163].

Finally, neither mutations of the late domain motifs altered the assembly rates [13], nor did protein labelling influence the time necessary for Gag accumulation on the PM [11]. Conversely, in good agreement with previous biochemical data [26], Gag proteins defective in the CA domain failed to retain viral RNA, which tended to dissociate from the membrane after about 8 min [159].

Similarly, to what was observed for HIV-1, TIRF microscopy on live cells showed that FIV gRNA, in the absence of Gag, moves in and out of the proximity of the PM, remaining therefore at those sites for no longer than a few seconds [114,162]. Conversely, when Gag is expressed, the genome accumulates at the PM. HIV-1 and FIV genomes co-expressed with the corresponding Gag mutants impaired in PM binding were not detectable at the PM, which provides additional evidence that Gag is responsible for anchoring the genomes at the assembly sites [10,114,164]. Those specific locations where HIV-1 assembly is initiated are enriched by lipid rafts, cholesterol and tetraspannines (reviewed in [36,165]). However, retroviruses developed multiple ways to assemble, and thus many membrane platforms give rise to multiple consecutive budding events of RSV differently from the mostly unique sites observed for MLV and HIV-1 [70,161]. TIRF analysis on individual assembling VLPs from the time of nucleation to the recruitment of VPS4, an ESCRT component which marks the final stage of VLP assembly, showed that VLPs can be paused (from 2 to 20 min) at various stages [163]. The cellular ESCRT machinery indeed corresponds to the molecular signature necessary for the separation of nascent VLPs from the host cell (reviewed in [166,167,168]). Finally, imaging HIV-1 particles generated with a Gag fused to pHluorin, (a GFP variant whose fluorescence is diminished at acidic pH) allowed for the observation that it takes about 30 min for virions to disconnect from the cytosol [11], which is in rather good agreement with data from Lamb et al. that showed that the release occurred with an average delay of about 25 min after the completion of the Gag assembly [13].

## 9. Concluding Remarks

In these last decades, the development of bioimaging tools allowed the visualization of retroviral genesis and led to a deeper quantitative analysis of the molecular mechanisms regulating gRNA packaging in living cells. As a result, instead of the differences among retroviruses, the recruitment of gRNA was overall observed to be driven by a small number of Gag molecules at different cellular sites (as cytoplasm or nucleus) depending on the retrovirus. Similarly, these techniques allowed for the monitoring of the time-dependent intracellular localization of viral components and the order of the events occurring at the viral budding sites. Indeed, the elaboration of these approaches addressed many important questions, although studies to this point have been limited to a few retroviruses, and there remain several open questions in the biology of retroviral assembly. Also, a number of cellular factors were observed to interact with HIV-1 Gag (e.g., APOBEC3G, Nedd4-1, Tsg101, Alix, ABCE1, Actin; for a review see [169]), and to be specifically encapsidated into the viral particles (e.g., Staufen, RNA 7SL) [170,171,172,173]. However, despite the plethora of studies on this topic, the actual implication of those molecules in HIV-1 assembly and replication still needs to be confirmed. With further technological and conceptual development, advancements in our understanding of the mechanisms leading to retroviral gRNA packaging and to their assembly awaits. In this vein the development of super-resolution techniques providing a resolution close to the molecular scale such as stimulated emission depletion (STED) [174], stochastic optical reconstruction microscopy (STORM) [175] and photo-activated localization microscopy (PALM) [176] would be determinant. These methods, that allow for penetration beyond the diffraction limit of conventional optical techniques and that achieve resolutions of about 20 nm in the focal plane (for reviews see [177,178]), would thus contribute to the clarity of our picture of the last phases of the retroviral life cycle and provide the experimental basis necessary to interfere with viral replication.

## Figures and Tables

**Table 1:** An overview of the advantages and drawbacks of the different methods used to monitor retroviral Gag-gRNA complexes in cells.

Method	Advantages	Disadvantages
Atomic Force Microscopy (AM)	Appropriate to measure the distortion of the membrane (resolution <1 nm up to 1µm).	Technically limited time resolution of several minutes per frame.
Electron Microscopy (EM)	Resolution of finer spatial detail compared to the classical microscopy (0.2 nm).	Cells are fixed, and temporal resolution is lost.
Confocal Microscopy	Suitable method to investigate processes that are not limited to the PM (resolution 180 nm laterally and 500 nm axially).	Low signal-to-noise ratio could require stronger illumination resulting in photobleached samples.
FLIM-FRET	Only the measurement of donor lifetime is required and acceptors with poor quantum yield can be used. Less excitation is required because of wider emission filters.	Careful measure of lifetime for the donor without the acceptor is required for accurate calibration.
Fluorescence Fluctuation Spectroscopy (FFS)	Ensemble of microscopy tools (e.g., FCCS and RICS) appropriate to analyze biomolecular dynamics, interactions, and structural changes in living cells.	Highly stable light source is necessary. Cumulative effects of photobleaching are possible. Analysis of molecules with different diffusive properties, as it can be the case of Gag, is complicated by the relative excitation intensities, different diffusion times, and the number of diffusing molecules for each population.
FRAP	Suitable method for determining the kinetics of diffusion in cells to study cellular membrane diffusion and membrane anchoring.	The estimation of intrinsic photobleaching. The precise identification of several mobile species corresponding to various degrees of oligomerization or having different interactions with the membranes, can be difficult.
TIRF	Accurate determination of axial position within ∼200 nm of the specimen surface. Proper to study events near to the PM as the retroviral assembly sites. It displays good signal to noise ratio to allow quantification of the assembly of individual HIV-1 particles.	The fluorescence signal can be affected by azimuthal movement of the VLP.

## Data Availability

Not applicable.

## References

[1] Mailler E., Bernacchi S., Marquet R., Paillart J.-C., Vivet-Boudou V., Smyth R. (2016). The Life-Cycle of the HIV-1 Gag–RNA Complex. Viruses.

[2] Rein A., Datta S.A.K., Jones C.P., Musier-Forsyth K. (2011). Diverse Interactions of Retroviral Gag Proteins with RNAs. Trends Biochem. Sci..

[3] Olson E.D., Musier-Forsyth K. (2019). Retroviral Gag Protein-RNA Interactions: Implications for Specific Genomic RNA Packaging and Virion Assembly. Semin. Cell Dev. Biol..

[4] Dubois N., Marquet R., Paillart J.-C., Bernacchi S. (2018). Retroviral RNA Dimerization: From Structure to Functions. Front. Microbiol..

[5] Lever A.M.L. (2000). HIV RNA Packaging and Lentivirus-Based Vectors. Advances in Pharmacology.

[6] Comas-Garcia M., Davis S., Rein A. (2016). On the Selective Packaging of Genomic RNA by HIV-1. Viruses.

[7] D’Souza V., Summers M.F. (2005). How Retroviruses Select Their Genomes. Nat. Rev. Microbiol..

[8] Rizvi T.A., Schmidt R.D., Lew K.A. (1997). Mason–Pfizer Monkey Virus (MPMV) Constitutive Transport Element (CTE) Functions in a Position-Dependent Manner. Virology.

[9] Jouvenet N., Neil S.J.D., Bess C., Johnson M.C., Virgen C.A., Simon S.M., Bieniasz P.D. (2006). Plasma Membrane Is the Site of Productive HIV-1 Particle Assembly. PLoS Biol..

[10] Jouvenet N., Simon S.M., Bieniasz P.D. (2009). Imaging the Interaction of HIV-1 Genomes and Gag during Assembly of Individual Viral Particles. PNAS.

[11] Jouvenet N., Bieniasz P.D., Simon S.M. (2008). Imaging the Biogenesis of Individual HIV-1 Virions in Live Cells. Nature.

[12] Chen J., Rahman S.A., Nikolaitchik O.A., Grunwald D., Sardo L., Burdick R.C., Plisov S., Liang E., Tai S., Pathak V.K. (2016). HIV-1 RNA Genome Dimerizes on the Plasma Membrane in the Presence of Gag Protein. Proc. Natl. Acad. Sci. USA.

[13] Ivanchenko S., Godinez W.J., Lampe M., Kräusslich H.-G., Eils R., Rohr K., Bräuchle C., Müller B., Lamb D.C. (2009). Dynamics of HIV-1 Assembly and Release. PLoS Pathog..

[14] Hendrix J., Baumgärtel V., Schrimpf W., Ivanchenko S., Digman M.A., Gratton E., Kräusslich H.-G., Müller B., Lamb D.C. (2015). Live-Cell Observation of Cytosolic HIV-1 Assembly Onset Reveals RNA-Interacting Gag Oligomers. J. Cell Biol..

[15] Sardo L., Hatch S.C., Chen J., Nikolaitchik O., Burdick R.C., Chen D., Westlake C.J., Lockett S., Pathak V.K., Hu W.-S. (2015). Dynamics of HIV-1 RNA Near the Plasma Membrane during Virus Assembly. J. Virol..

[16] Hubner W., Chen P., Portillo A.D., Liu Y., Gordon R.E., Chen B.K. (2007). Sequence of Human Immunodeficiency Virus Type 1 (HIV-1) Gag Localization and Oligomerization Monitored with Live Confocal Imaging of a Replication-Competent, Fluorescently Tagged HIV-1. J. Virol..

[17] Larson D.R., Ma Y.M., Vogt V.M., Webb W.W. (2003). Direct Measurement of Gag–Gag Interaction during Retrovirus Assembly with FRET and Fluorescence Correlation Spectroscopy. J. Cell. Biol..

[18] Arhel N., Genovesio A., Kim K.-A., Miko S., Perret E., Olivo-Marin J.-C., Shorte S., Charneau P. (2006). Quantitative Four-Dimensional Tracking of Cytoplasmic and Nuclear HIV-1 Complexes. Nat. Methods.

[19] McDonald D. (2006). The inside Track on HIV. Nat. Methods.

[20] Rudner L., Nydegger S., Coren L.V., Nagashima K., Thali M., Ott D.E. (2005). Dynamic Fluorescent Imaging of Human Immunodeficiency Virus Type 1 Gag in Live Cells by Biarsenical Labeling. J. Virol..

[21] Boutant E., Bonzi J., Anton H., Nasim M.B., Cathagne R., Réal E., Dujardin D., Carl P., Didier P., Paillart J.-C. (2020). Zinc Fingers in HIV-1 Gag Precursor Are Not Equivalent for GRNA Recruitment at the Plasma Membrane. Biophys. J..

[22] Chen J., Grunwald D., Sardo L., Galli A., Plisov S., Nikolaitchik O.A., Chen D., Lockett S., Larson D.R., Pathak V.K. (2014). Cytoplasmic HIV-1 RNA Is Mainly Transported by Diffusion in the Presence or Absence of Gag Protein. Proc. Natl. Acad. Sci. USA.

[23] Ferrer M., Clerté C., Chamontin C., Basyuk E., Lainé S., Hottin J., Bertrand E., Margeat E., Mougel M. (2016). Imaging HIV-1 RNA Dimerization in Cells by Multicolor Super-Resolution and Fluctuation Microscopies. Nucleic Acids Res..

[24] Carlson L.-A., Briggs J.A.G., Glass B., Riches J.D., Simon M.N., Johnson M.C., Müller B., Grünewald K., Kräusslich H.-G. (2008). Three-Dimensional Analysis of Budding Sites and Released Virus Suggests a Revised Model for HIV-1 Morphogenesis. Cell Host Microbe.

[25] Kutluay S.B., Zang T., Blanco-Melo D., Powell C., Jannain D., Errando M., Bieniasz P.D. (2014). Global Changes in the RNA Binding Specificity of HIV-1 Gag Regulate Virion Genesis. Cell.

[26] Kutluay S.B., Bieniasz P.D. (2010). Analysis of the Initiating Events in HIV-1 Particle Assembly and Genome Packaging. PLoS Pathog..

[27] Bernacchi S., Abd El-Wahab E.W., Dubois N., Hijnen M., Smyth R.P., Mak J., Marquet R., Paillart J.-C. (2017). HIV-1 Pr55 ^Gag^ Binds Genomic and Spliced RNAs with Different Affinity and Stoichiometry. RNA Biol..

[28] Briggs J.A.G., Simon M.N., Gross I., Kräusslich H.-G., Fuller S.D., Vogt V.M., Johnson M.C. (2004). The Stoichiometry of Gag Protein in HIV-1. Nat. Struct. Mol. Biol..

[29] Freed E.O. (2015). HIV-1 Assembly, Release and Maturation. Nat. Rev. Microbiol..

[30] Bussienne C., Marquet R., Paillart J.-C., Bernacchi S. (2021). Post-Translational Modifications of Retroviral HIV-1 Gag Precursors: An Overview of Their Biological Role. IJMS.

[31] Zhou W., Resh M.D. (1996). Differential Membrane Binding of the Human Immunodeficiency Virus Type 1 Matrix Protein. J. Virol..

[32] Paillart J.-C., Göttlinger H.G. (1999). Opposing Effects of Human Immunodeficiency Virus Type 1 Matrix Mutations Support a Myristyl Switch Model of Gag Membrane Targeting. J. Virol..

[33] Tang C., Loeliger E., Luncsford P., Kinde I., Beckett D., Summers M.F. (2004). Entropic Switch Regulates Myristate Exposure in the HIV-1 Matrix Protein. Proc. Natl. Acad. Sci. USA.

[34] Sandefur S., Smith R.M., Varthakavi V., Spearman P. (2000). Mapping and Characterization of the N-Terminal I Domain of Human Immunodeficiency Virus Type 1 Pr55 ^Gag^. J. Virol..

[35] Ono A., Freed E.O. (2004). Cell-Type-Dependent Targeting of Human Immunodeficiency Virus Type 1 Assembly to the Plasma Membrane and the Multivesicular Body. J. Virol..

[36] Chukkapalli V., Ono A. (2011). Molecular Determinants That Regulate Plasma Membrane Association of HIV-1 Gag. J. Mol. Biol..

[37] Chukkapalli V., Hogue I.B., Boyko V., Hu W.-S., Ono A. (2008). Interaction between the Human Immunodeficiency Virus Type 1 Gag Matrix Domain and Phosphatidylinositol-(4,5)-Bisphosphate Is Essential for Efficient Gag Membrane Binding. J. Virol..

[38] Saad J.S., Miller J., Tai J., Kim A., Ghanam R.H., Summers M.F. (2006). Structural Basis for Targeting HIV-1 Gag Proteins to the Plasma Membrane for Virus Assembly. Proc. Natl. Acad. Sci. USA.

[39] Jones C.P., Datta S.A.K., Rein A., Rouzina I., Musier-Forsyth K. (2011). Matrix Domain Modulates HIV-1 Gag’s Nucleic Acid Chaperone Activity via Inositol Phosphate Binding. J. Virol..

[40] Todd G.C., Duchon A., Inlora J., Olson E.D., Musier-Forsyth K., Ono A. (2017). Inhibition of HIV-1 Gag–Membrane Interactions by Specific RNAs. RNA.

[41] Chukkapalli V., Oh S.J., Ono A. (2010). Opposing Mechanisms Involving RNA and Lipids Regulate HIV-1 Gag Membrane Binding through the Highly Basic Region of the Matrix Domain. Proc. Natl. Acad. Sci. USA.

[42] Bieniasz P., Telesnitsky A. (2018). Multiple, Switchable Protein:RNA Interactions Regulate Human Immunodeficiency Virus Type 1 Assembly. Annu. Rev. Virol..

[43] Munro J.B., Nath A., Färber M., Datta S.A.K., Rein A., Rhoades E., Mothes W. (2014). A Conformational Transition Observed in Single HIV-1 Gag Molecules during *In Vitro* Assembly of Virus-Like Particles. J. Virol..

[44] Obr M., Ricana C.L., Nikulin N., Feathers J.-P.R., Klanschnig M., Thader A., Johnson M.C., Vogt V.M., Schur F.K.M., Dick R.A. (2021). Structure of the Mature Rous Sarcoma Virus Lattice Reveals a Role for IP6 in the Formation of the Capsid Hexamer. Nat. Commun..

[45] Schur F.K.M., Dick R.A., Hagen W.J.H., Vogt V.M., Briggs J.A.G. (2015). The Structure of Immature Virus-Like Rous Sarcoma Virus Gag Particles Reveals a Structural Role for the P10 Domain in Assembly. J. Virol..

[46] Gamble T.R., Yoo S., Vajdos F.F., von Schwedler U.K., Worthylake D.K., Wang H., McCutcheon J.P., Sundquist W.I., Hill C.P. (1997). Structure of the Carboxyl-Terminal Dimerization Domain of the HIV-1 Capsid Protein. Science.

[47] Von Schwedler U.K., Stray K.M., Garrus J.E., Sundquist W.I. (2003). Functional Surfaces of the Human Immunodeficiency Virus Type 1 Capsid Protein. JVI.

[48] Dorfman T., Bukovsky A., Ohagen A., Höglund S., Göttlinger H.G. (1994). Functional Domains of the Capsid Protein of Human Immunodeficiency Virus Type 1. J. Virol..

[49] Accola M.A., Strack B., Göttlinger H.G. (2000). Efficient Particle Production by Minimal Gag Constructs Which Retain the Carboxy-Terminal Domain of Human Immunodeficiency Virus Type 1 Capsid-P2 and a Late Assembly Domain. J. Virol..

[50] Ma Y.M., Vogt V.M. (2002). Rous Sarcoma Virus Gag Protein-Oligonucleotide Interaction Suggests a Critical Role for Protein Dimer Formation in Assembly. J. Virol..

[51] Fisher R.J. (2006). Complex Interactions of HIV-1 Nucleocapsid Protein with Oligonucleotides. Nucleic Acids Res..

[52] Houzet L., Morichaud Z., Didierlaurent L., Muriaux D., Darlix J.-L., Mougel M. (2008). Nucleocapsid Mutations Turn HIV-1 into a DNA-Containing Virus. Nucleic Acids Res..

[53] Didierlaurent L., Houzet L., Morichaud Z., Darlix J.-L., Mougel M. (2008). The Conserved N-Terminal Basic Residues and Zinc-Finger Motifs of HIV-1 Nucleocapsid Restrict the Viral CDNA Synthesis during Virus Formation and Maturation. Nucleic Acids Res..

[54] Sun M., Grigsby I.F., Gorelick R.J., Mansky L.M., Musier-Forsyth K. (2014). Retrovirus-Specific Differences in Matrix and Nucleocapsid Protein-Nucleic Acid Interactions: Implications for Genomic RNA Packaging. J. Virol..

[55] Dick R.A., Datta S.A.K., Nanda H., Fang X., Wen Y., Barros M., Wang Y.-X., Rein A., Vogt V.M. (2015). Hydrodynamic and Membrane Binding Properties of Purified Rous Sarcoma Virus Gag Protein. J. Virol..

[56] Zábranský A., Hoboth P., Hadravová R., Štokrová J., Sakalian M., Pichová I. (2010). The Noncanonical Gag Domains P8 and n Are Critical for Assembly and Release of Mouse Mammary Tumor Virus. J. Virol..

[57] Abdusetir Cerfoglio J.C., González S.A., Affranchino J.L. (2014). Structural Elements in the Gag Polyprotein of Feline Immunodeficiency Virus Involved in Gag Self-Association and Assembly. J. Gen. Virol..

[58] Parent L.J., Bennett R.P., Craven R.C., Nelle T.D., Krishna N.K., Bowzard J.B., Wilson C.B., Puffer B.A., Montelaro R.C., Wills J.W. (1995). Positionally Independent and Exchangeable Late Budding Functions of the Rous Sarcoma Virus and Human Immunodeficiency Virus Gag Proteins. J. Virol..

[59] Yasuda J., Hunter E. (1998). A Proline-Rich Motif (PPPY) in the Gag Polyprotein of Mason-Pfizer Monkey Virus Plays a Maturation-Independent Role in Virion Release. J. Virol..

[60] Welker L., Paillart J.-C., Bernacchi S. (2021). Importance of Viral Late Domains in Budding and Release of Enveloped RNA Viruses. Viruses.

[61] Mendonça L., Sun D., Ning J., Liu J., Kotecha A., Olek M., Frosio T., Fu X., Himes B.A., Kleinpeter A.B. (2021). CryoET Structures of Immature HIV Gag Reveal Six-Helix Bundle. Commun. Biol..

[62] Wiegers K., Rutter G., Kottler H., Tessmer U., Hohenberg H., Kräusslich H.-G. (1998). Sequential Steps in Human Immunodeficiency Virus Particle Maturation Revealed by Alterations of Individual Gag Polyprotein Cleavage Sites. J. Virol..

[63] Roy B.B., Russell R.S., Turner D., Liang C. (2006). The T12I Mutation within the SP1 Region of Gag Restricts Packaging of Spliced Viral RNA into Human Immunodeficiency Virus Type 1 with Mutated RNA Packaging Signals and Mutated Nucleocapsid Sequence. Virology.

[64] Russell R.S., Roldan A., Detorio M., Hu J., Wainberg M.A., Liang C. (2003). Effects of a Single Amino Acid Substitution within Thep2 Region of Human Immunodeficiency Virus Type 1 on Packagingof Spliced ViralRNA. J. Virol..

[65] Wanaguru M., Barry D.J., Benton D.J., O’Reilly N.J., Bishop K.N. (2018). Murine Leukemia Virus P12 Tethers the Capsid-Containing Pre-Integration Complex to Chromatin by Binding Directly to Host Nucleosomes in Mitosis. PLoS Pathog..

[66] Tanwar H.S., Khoo K.K., Garvey M., Waddington L., Leis A., Hijnen M., Velkov T., Dumsday G.J., McKinstry W.J., Mak J. (2017). The Thermodynamics of Pr55Gag-RNA Interaction Regulate the Assembly of HIV. PLoS Pathog..

[67] Dubois N., Khoo K.K., Ghossein S., Seissler T., Wolff P., McKinstry W.J., Mak J., Paillart J.-C., Marquet R., Bernacchi S. (2018). The C-Terminal P6 Domain of the HIV-1 Pr55 ^Gag^ Precursor Is Required for Specific Binding to the Genomic RNA. RNA Biol..

[68] Müller B., Daecke J., Fackler O.T., Dittmar M.T., Zentgraf H., Kräusslich H.-G. (2004). Construction and Characterization of a Fluorescently Labeled Infectious Human Immunodeficiency Virus Type 1 Derivative. J. Virol..

[69] Pornillos O., Higginson D.S., Stray K.M., Fisher R.D., Garrus J.E., Payne M., He G.-P., Wang H.E., Morham S.G., Sundquist W.I. (2003). HIV Gag Mimics the Tsg101-Recruiting Activity of the Human Hrs Protein. J. Cell Biol..

[70] Larson D.R., Johnson M.C., Webb W.W., Vogt V.M. (2005). Visualization of Retrovirus Budding with Correlated Light and Electron Microscopy. Proc. Natl. Acad. Sci. USA.

[71] Wachter R. (2017). Photoconvertible Fluorescent Proteins and the Role of Dynamics in Protein Evolution. IJMS.

[72] Shaner N.C., Steinbach P.A., Tsien R.Y. (2005). A Guide to Choosing Fluorescent Proteins. Nat. Methods.

[73] Wiedenmann J., Oswald F., Nienhaus G.U. (2009). Fluorescent Proteins for Live Cell Imaging: Opportunities, Limitations, and Challenges. IUBMB Life.

[74] Griffin B.A., Adams S.R., Tsien R.Y. (1998). Specific Covalent Labeling of Recombinant Protein Molecules Inside Live Cells. Science.

[75] Lee Y.-M., Yu X.-F. (1998). Identification and Characterization of Virus Assembly Intermediate Complexes in HIV-1-Infected CD4+T Cells. Virology.

[76] Nermut M.V., Fassati A. (2003). Structural Analyses of Purified Human Immunodeficiency Virus Type 1 Intracellular Reverse Transcription Complexes. J. Virol..

[77] Gomez C.Y., Hope T.J. (2006). Mobility of Human Immunodeficiency Virus Type 1 Pr55 ^Gag^ in Living Cells. J. Virol..

[78] Hogue I.B., Hoppe A., Ono A. (2009). Quantitative Fluorescence Resonance Energy Transfer Microscopy Analysis of the Human Immunodeficiency Virus Type 1 Gag-Gag Interaction: Relative Contributions of the CA and NC Domains and Membrane Binding. J. Virol..

[79] Alfadhli A., Dhenub T.C., Still A., Barklis E. (2005). Analysis of Human Immunodeficiency Virus Type 1 Gag Dimerization-Induced Assembly. J. Virol..

[80] Johnson M.C., Scobie H.M., Ma Y.M., Vogt V.M. (2002). Nucleic Acid-Independent Retrovirus Assembly Can Be Driven by Dimerization. J. Virol..

[81] Ma Y.M., Vogt V.M. (2004). Nucleic Acid Binding-Induced Gag Dimerization in the Assembly of Rous Sarcoma Virus Particles In Vitro. J. Virol..

[82] Roldan A., Russell R.S., Marchand B., Götte M., Liang C., Wainberg M.A. (2004). In Vitro Identification and Characterization of an Early Complex Linking HIV-1 Genomic RNA Recognition and Pr55Gag Multimerization. J. Biol. Chem..

[83] Guo X., Roy B.B., Hu J., Roldan A., Wainberg M.A., Liang C. (2005). The R362A Mutation at the C-Terminus of CA Inhibits Packaging of Human Immunodeficiency Virus Type 1 RNA. Virology.

[84] Kaye J.F., Lever A.M.L. (1998). Nonreciprocal Packaging of Human Immunodeficiency Virus Type 1 and Type 2 RNA: A Possible Role for the P2 Domain of Gag in RNA Encapsidation. J. Virol..

[85] Carlson L.-A., Bai Y., Keane S.C., Doudna J.A., Hurley J.H. (2016). Reconstitution of Selective HIV-1 RNA Packaging in Vitro by Membrane-Bound Gag Assemblies. eLife.

[86] Fogarty K.H., Chen Y., Grigsby I.F., Macdonald P.J., Smith E.M., Johnson J.L., Rawson J.M., Mansky L.M., Mueller J.D. (2011). Characterization of Cytoplasmic Gag-Gag Interactions by Dual-Color Z-Scan Fluorescence Fluctuation Spectroscopy. Biophys. J..

[87] Pillai V.N., Ali L.M., Prabhu S.G., Krishnan A., Chameettachal A., Pitchai F.N.N., Mustafa F., Rizvi T.A. (2021). A Stretch of Unpaired Purines in the Leader Region of Simian Immunodeficiency Virus (SIV) Genomic RNA Is Critical for Its Packaging into Virions. J. Mol. Biol..

[88] Ali L.M., Pitchai F.N.N., Vivet-Boudou V., Chameettachal A., Jabeen A., Pillai V.N., Mustafa F., Marquet R., Rizvi T.A. (2020). Role of Purine-Rich Regions in Mason-Pfizer Monkey Virus (MPMV) Genomic RNA Packaging and Propagation. Front. Microbiol..

[89] Jaballah S.A., Aktar S.J., Ali J., Phillip P.S., Al Dhaheri N.S., Jabeen A., Rizvi T.A. (2010). A G–C-Rich Palindromic Structural Motif and a Stretch of Single-Stranded Purines Are Required for Optimal Packaging of Mason–Pfizer Monkey Virus (MPMV) Genomic RNA. J. Mol. Biol..

[90] Houzet L., Paillart J.C., Smagulova F., Maurel S., Morichaud Z., Marquet R., Mougel M. (2007). HIV Controls the Selective Packaging of Genomic, Spliced Viral and Cellular RNAs into Virions through Different Mechanisms. Nucleic Acids Res..

[91] Smyth R.P., Despons L., Huili G., Bernacchi S., Hijnen M., Mak J., Jossinet F., Weixi L., Paillart J.-C., von Kleist M. (2015). Mutational Interference Mapping Experiment (MIME) for Studying RNA Structure and Function. Nat. Methods.

[92] Tounekti N., Mougel M., Roy C., Marquet R., Darlix J.-L., Paoletti J., Ehresmann B., Ehresmann C. (1992). Effect of Dimerization on the Conformation of the Encapsidation Psi Domain of Moloney Murine Leukemia Virus RNA. J. Mol. Biol..

[93] Mougel M., Barklis E. (1997). A Role for Two Hairpin Structures as a Core RNA Encapsidation Signal in Murine Leukemia Virus Virions. J. Virol..

[94] Oroudjev E.M., Kang P.C.E., Kohlstaedt L.A. (1999). An Additional Dimer Linkage Structure in Moloney Murine Leukemia Virus RNA 1 1Edited by D. E. Draper. J. Mol. Biol..

[95] Ly H., Parslow T.G. (2002). Bipartite Signal for Genomic RNA Dimerization in Moloney Murine Leukemia Virus. J. Virol..

[96] Liu S., Kaddis Maldonado R., Rye-McCurdy T., Binkley C., Bah A., Chen E.C., Rice B.L., Parent L.J., Musier-Forsyth K. (2020). Rous Sarcoma Virus Genomic RNA Dimerization Capability In Vitro Is Not a Prerequisite for Viral Infectivity. Viruses.

[97] Lever A., Gottlinger H., Haseltine W., Sodroski J. (1989). Identification of a Sequence Required for Efficient Packaging of Human Immunodeficiency Virus Type 1 RNA into Virions. J. Virol..

[98] Clever J., Sassetti C., Parslow T.G. (1995). RNA Secondary Structure and Binding Sites for Gag Gene Products in the 5’ Packaging Signal of Human Immunodeficiency Virus Type 1. J. Virol..

[99] Laughrea M., Jetté L., Mak J., Kleiman L., Liang C., Wainberg M.A. (1997). Mutations in the Kissing-Loop Hairpin of Human Immunodeficiency Virus Type 1 Reduce Viral Infectivity as Well as Genomic RNA Packaging and Dimerization. J. Virol..

[100] Ennifar E., Walter P., Ehresmann B., Ehresmann C., Dumas P. (2001). Crystal Structures of Coaxially Stacked Kissing Complexes of the HIV-1 RNA Dimerization Iniziatin Site. Nat. Struct. Biol..

[101] Ennifar E., Carpentier P., Ferrer J.L., Walter P., Dumas P. (2002). X-Ray-Induced Debromination of Nucleic Acids at the Br K Absorption Edge and Implications for MAD Phasing. Acta Cryst. D Biol. Cryst..

[102] Skripkin E., Paillart J.C., Marquet R., Ehresmann B., Ehresmann C. (1994). Identification of the Primary Site of the Human Immunodeficiency Virus Type 1 RNA Dimerization in Vitro. Proc. Natl. Acad. Sci. USA.

[103] Paillart J.C., Skripkin E., Ehresmann B., Ehresmann C., Marquet R. (1996). A Loop-Loop “Kissing” Complex Is the Essential Part of the Dimer Linkage of Genomic HIV-1 RNA. Proc. Natl. Acad. Sci. USA.

[104] Weixlbaumer A. (2004). Determination of Thermodynamic Parameters for HIV DIS Type Loop-Loop Kissing Complexes. Nucleic Acids Res..

[105] Chameettachal A., Vivet-Boudou V., Pitchai F.N.N., Pillai V.N., Ali L.M., Krishnan A., Bernacchi S., Mustafa F., Marquet R., Rizvi T.A. (2021). A Purine Loop and the Primer Binding Site Are Critical for the Selective Encapsidation of Mouse Mammary Tumor Virus Genomic RNA by Pr77Gag. Nucleic Acids Res..

[106] Muriaux D., Fossé P., Paoletti J. (1996). A Kissing Complex Together with a Stable Dimer Is Involved in the HIV-1 _Lai_ RNA Dimerization Process in Vitro. Biochemistry.

[107] Bernacchi S., Ennifar E., Tóth K., Walter P., Langowski J., Dumas P. (2005). Mechanism of Hairpin-Duplex Conversion for the HIV-1 Dimerization Initiation Site. J. Biol. Chem..

[108] Levin J.G., Guo J., Rouzina I., Musier-Forsyth K. (2005). Nucleic Acid Chaperone Activity of HIV-1 Nucleocapsid Protein: Critical Role in Reverse Transcription and Molecular Mechanism. Progress in Nucleic Acid Research and Molecular Biology.

[109] Rist M.J., Marino J.P. (2002). Mechanism of Nucleocapsid Protein Catalyzed Structural Isomerization of the Dimerization Initiation Site of HIV-1. Biochemistry.

[110] Brigham B.S., Kitzrow J.P., Reyes J.-P.C., Musier-Forsyth K., Munro J.B. (2019). Intrinsic Conformational Dynamics of the HIV-1 Genomic RNA 5′UTR. Proc. Natl. Acad. Sci. USA.

[111] Lu K., Heng X., Garyu L., Monti S., Garcia E.L., Kharytonchyk S., Dorjsuren B., Kulandaivel G., Jones S., Hiremath A. (2011). NMR Detection of Structures in the HIV-1 5’-Leader RNA That Regulate Genome Packaging. Science.

[112] Tran T., Liu Y., Marchant J., Monti S., Seu M., Zaki J., Yang A.L., Bohn J., Ramakrishnan V., Singh R. (2015). Conserved Determinants of Lentiviral Genome Dimerization. Retrovirology.

[113] Kalloush R.M., Vivet-Boudou V., Ali L.M., Pillai V.N., Mustafa F., Marquet R., Rizvi T.A. (2019). Stabilizing Role of Structural Elements within the 5´ Untranslated Region (UTR) and Gag Sequences in Mason-Pfizer Monkey Virus (MPMV) Genomic RNA Packaging. RNA Biol..

[114] Kemler I., Meehan A., Poeschla E.M. (2010). Live-Cell Coimaging of the Genomic RNAs and Gag Proteins of Two Lentiviruses. J. Virol..

[115] Pocock G.M., Becker J.T., Swanson C.M., Ahlquist P., Sherer N.M. (2016). HIV-1 and M-PMV RNA Nuclear Export Elements Program Viral Genomes for Distinct Cytoplasmic Trafficking Behaviors. PLoS Pathog..

[116] Becker J.T., Sherer N.M. (2017). Subcellular Localization of HIV-1 *Gag-Pol* MRNAs Regulates Sites of Virion Assembly. J. Virol..

[117] Chen J., Nikolaitchik O., Singh J., Wright A., Bencsics C.E., Coffin J.M., Ni N., Lockett S., Pathak V.K., Hu W.-S. (2009). High Efficiency of HIV-1 Genomic RNA Packaging and Heterozygote Formation Revealed by Single Virion Analysis. Proc. Natl. Acad. Sci. USA.

[118] St Johnston D. (2005). Moving Messages: The Intracellular Localization of MRNAs. Nat. Rev. Mol. Cell Biol..

[119] Fusco D., Bertrand E., Singer R.H., Jeanteur P. (2008). Imaging of Single MRNAs in the Cytoplasm of Living Cells. RNA Trafficking and Nuclear Structure Dynamics.

[120] Sinck L., Richer D., Howard J., Alexander M., Purcell D.F.J., Marquet R., Paillart J.-C. (2007). In Vitro Dimerization of Human Immunodeficiency Virus Type 1 (HIV-1) Spliced RNAs. RNA.

[121] Moore M.D., Nikolaitchik O.A., Chen J., Hammarskjöld M.-L., Rekosh D., Hu W.-S. (2009). Probing the HIV-1 Genomic RNA Trafficking Pathway and Dimerization by Genetic Recombination and Single Virion Analyses. PLoS Pathog..

[122] Maurel S., Houzet L., Garcia E.L., Telesnitsky A., Mougel M. (2007). Characterization of a Natural Heterodimer between MLV Genomic RNA and the SD′ Retroelement Generated by Alternative Splicing. RNA.

[123] Maurel S., Mougel M. (2010). Murine Leukemia Virus RNA Dimerization Is Coupled to Transcription and Splicing Processes. Retrovirology.

[124] Chen E.C., Maldonado R.J.K., Parent L.J. (2021). Visualizing Rous Sarcoma Virus Genomic RNA Dimerization in the Nucleus, Cytoplasm, and at the Plasma Membrane. Viruses.

[125] Basyuk E., Boulon S., Skou Pedersen F., Bertrand E., Vestergaard Rasmussen S. (2005). The Packaging Signal of MLV Is an Integrated Module That Mediates Intracellular Transport of Genomic RNAs. J. Mol. Biol..

[126] Smagulova F., Maurel S., Morichaud Z., Devaux C., Mougel M., Houzet L. (2005). The Highly Structured Encapsidation Signal of MuLV RNA Is Involved in the Nuclear Export of Its Unspliced RNA. J. Mol. Biol..

[127] Abd El-Wahab E.W., Smyth R.P., Mailler E., Bernacchi S., Vivet-Boudou V., Hijnen M., Jossinet F., Mak J., Paillart J.-C., Marquet R. (2014). Specific Recognition of the HIV-1 Genomic RNA by the Gag Precursor. Nat. Commun..

[128] Muriaux D., Darlix J.-L. (2010). Properties and Functions of the Nucleocapsid Protein in Virus Assembly. RNA Biol..

[129] Rein A. (2010). Nucleic Acid Chaperone Activity of Retroviral Gag Proteins. RNA Biol..

[130] Rein A., Henderson L.E., Levin J.G. (1998). Nucleic-Acid-Chaperone Activity of Retroviral Nucleocapsid Proteins: Significance for Viral Replication. Trends Biochem. Sci..

[131] Paillart J.C., Berthoux L., Ottmann M., Darlix J.L., Marquet R., Ehresmann B., Ehresmann C. (1996). A Dual Role of the Putative RNA Dimerization Initiation Site of Human Immunodeficiency Virus Type 1 in Genomic RNA Packaging and Proviral DNA Synthesis. J. Virol..

[132] Berkhout B., van Wamel J.L. (1996). Role of the DIS Hairpin in Replication of Human Immunodeficiency Virus Type 1. J. Virol..

[133] Chen J., Liu Y., Wu B., Nikolaitchik O.A., Mohan P.R., Chen J., Pathak V.K., Hu W.-S. (2020). Visualizing the Translation and Packaging of HIV-1 Full-Length RNA. Proc. Natl. Acad. Sci. USA.

[134] Parent L.J. (2011). New Insights into the Nuclear Localization of Retroviral Gag Proteins. Nucleus.

[135] Garbitt-Hirst R., Kenney S.P., Parent L.J. (2009). Genetic Evidence for a Connection between Rous Sarcoma Virus Gag Nuclear Trafficking and Genomic RNA Packaging. J. Virol..

[136] Gudleski N., Flanagan J.M., Ryan E.P., Bewley M.C., Parent L.J. (2010). Directionality of Nucleocytoplasmic Transport of the Retroviral Gag Protein Depends on Sequential Binding of Karyopherins and Viral RNA. Proc. Natl. Acad. Sci. USA.

[137] Kenney S.P., Lochmann T.L., Schmid C.L., Parent L.J. (2008). Intermolecular Interactions between Retroviral Gag Proteins in the Nucleus. J. Virol..

[138] Nash M.A., Meyer M.K., Decker G.L., Arlinghaus R.B. (1993). A Subset of Pr65gag Is Nucleus Associated in Murine Leukemia Virus-Infected Cells. J. Virol..

[139] Schliephake A.W., Rethwilm A. (1994). Nuclear Localization of Foamy Virus Gag Precursor Protein. J. Virol..

[140] Beyer A.R., Bann D.V., Rice B., Pultz I.S., Kane M., Goff S.P., Golovkina T.V., Parent L.J. (2013). Nucleolar Trafficking of the Mouse Mammary Tumor Virus Gag Protein Induced by Interaction with Ribosomal Protein L9. J. Virol..

[141] Cullen B.R. (2003). Nuclear MRNA Export: Insights from Virology. Trends Biochem. Sci..

[142] Butterfield-Gerson K.L., Scheifele L.Z., Ryan E.P., Hopper A.K., Parent L.J. (2006). Importin-β Family Members Mediate Alpharetrovirus Gag Nuclear Entry via Interactions with Matrix and Nucleocapsid. J. Virol..

[143] Kaddis Maldonado R., Parent L. (2016). Orchestrating the Selection and Packaging of Genomic RNA by Retroviruses: An Ensemble of Viral and Host Factors. Viruses.

[144] Yu F., Joshi S.M., Ma Y.M., Kingston R.L., Simon M.N., Vogt V.M. (2001). Characterization of Rous Sarcoma Virus Gag Particles Assembled In Vitro. J. Virol..

[145] Scheifele L.Z., Ryan E.P., Parent L.J. (2005). Detailed Mapping of the Nuclear Export Signal in the Rous Sarcoma Virus Gag Protein. J. Virol..

[146] Scheifele L.Z., Garbitt R.A., Rhoads J.D., Parent L.J. (2002). Nuclear Entry and CRM1-Dependent Nuclear Export of the Rous Sarcoma Virus Gag Polyprotein. PNAS.

[147] Maldonado R.J.K., Rice B., Chen E.C., Tuffy K.M., Chiari E.F., Fahrbach K.M., Hope T.J., Parent L.J. (2020). Visualizing Association of the Retroviral Gag Protein with Unspliced Viral RNA in the Nucleus. mBio.

[148] Hernandez F.P., Sandri-Goldin R.M. (2011). Bimolecular Fluorescence Complementation Analysis to Reveal Protein Interactions in Herpes Virus Infected Cells. Methods.

[149] Tuffy K.M., Maldonado R.J.K., Chang J., Rosenfeld P., Cochrane A., Parent L.J. (2020). HIV-1 Gag Forms Ribonucleoprotein Complexes with Unspliced Viral RNA at Transcription Sites. Viruses.

[150] Dupont S., Sharova N., DéHoratius C., Virbasius C.-M.A., Zhu X., Bukrinskaya A.G., Stevenson M., Green M.R. (1999). A Novel Nuclear Export Activity in HIV-1 Matrix Protein Required for Viral Replication. Nature.

[151] Poole E., Strappe P., Mok H.-P., Hicks R., Lever A.M.L. (2005). HIV-1 Gag-RNA Interaction Occurs at a Perinuclear/Centrosomal Site; Analysis by Confocal Microscopy and FRET: HIV-1 Gag-RNA Interaction Occurs in a Perinuclear Region. Traffic.

[152] Baumgärtel V., Müller B., Lamb D.C. (2012). Quantitative Live-Cell Imaging of Human Immunodeficiency Virus (HIV-1) Assembly. Viruses.

[153] Digman M.A., Stakic M., Gratton E. (2013). Raster Image Correlation Spectroscopy and Number and Brightness Analysis. Methods in Enzymology.

[154] Rhee S.S., Hunter E. (1987). Myristylation Is Required for Intracellular Transport but Not for Assembly of D-Type Retrovirus Capsids. J. Virol..

[155] Schultz A.M., Oroszlan S. (1983). In Vivo Modification of Retroviral Gag Gene-Encoded Polyproteins by Myristic Acid. J. Virol..

[156] Houzet L., Gay B., Morichaud Z., Briant L., Mougel M. (2006). Intracellular Assembly and Budding of the Murine Leukemia Virus in Infected Cells. Retrovirology.

[157] Blot V., Perugi F., Gay B., Prévost M.-C., Briant L., Tangy F., Abriel H., Staub O., Dokhélar M.-C., Pique C. (2004). Nedd4.1-Mediated Ubiquitination and Subsequent Recruitment of Tsg101 Ensure HTLV-1 Gag Trafficking towards the Multivesicular Body Pathway Prior to Virus Budding. J. Cell Sci..

[158] Basyuk E., Galli T., Mougel M., Blanchard J.-M., Sitbon M., Bertrand E. (2003). Retroviral Genomic RNAs Are Transported to the Plasma Membrane by Endosomal Vesicles. Dev. Cell.

[159] Jouvenet N., Lainé S., Pessel-Vivares L., Mougel M. (2011). Cell Biology of Retroviral RNA Packaging. RNA Biol..

[160] Welsch S., Keppler O.T., Habermann A., Allespach I., Krijnse-Locker J., Kräusslich H.-G. (2007). HIV-1 Buds Predominantly at the Plasma Membrane of Primary Human Macrophages. PLoS Pathog..

[161] Gladnikoff M., Rousso I. (2008). Directly Monitoring Individual Retrovirus Budding Events Using Atomic Force Microscopy. Biophys. J..

[162] Saffarian S., Kirchhausen T. (2008). Differential Evanescence Nanometry: Live-Cell Fluorescence Measurements with 10-Nm Axial Resolution on the Plasma Membrane. Biophys. J..

[163] Ku P.-I., Miller A.K., Ballew J., Sandrin V., Adler F.R., Saffarian S. (2013). Identification of Pauses during Formation of HIV-1 Virus Like Particles. Biophys. J..

[164] Kemler I., Barraza R., Poeschla E.M. (2002). Mapping the Encapsidation Determinants of Feline Immunodeficiency Virus. J. Virol..

[165] Kerviel A., Thomas A., Chaloin L., Favard C., Muriaux D. (2013). Virus Assembly and Plasma Membrane Domains: Which Came First?. Virus Res..

[166] Weiss E.R., Göttlinger H. (2011). The Role of Cellular Factors in Promoting HIV Budding. J. Mol. Biol..

[167] Hurley J.H., Boura E., Carlson L.-A., Różycki B. (2010). Membrane Budding. Cell.

[168] Martin-Serrano J., Neil S.J.D. (2011). Host Factors Involved in Retroviral Budding and Release. Nat. Rev. Microbiol..

[169] Klingler J., Anton H., Réal E., Zeiger M., Moog C., Mély Y., Boutant E. (2020). How HIV-1 Gag Manipulates Its Host Cell Proteins: A Focus on Interactors of the Nucleocapsid Domain. Viruses.

[170] Mouland A.J., Mercier J., Luo M., Bernier L., DesGroseillers L., Cohen É.A. (2000). The Double-Stranded RNA-Binding Protein Staufen Is Incorporated in Human Immunodeficiency Virus Type 1: Evidence for a Role in Genomic RNA Encapsidation. J. Virol..

[171] Chatel-Chaix L., Clément J.-F., Martel C., Bériault V., Gatignol A., DesGroseillers L., Mouland A.J. (2004). Identification of Staufen in the Human Immunodeficiency Virus Type 1 Gag Ribonucleoprotein Complex and a Role in Generating Infectious Viral Particles. Mol. Cell Biol..

[172] Itano M.S., Arnion H., Wolin S.L., Simon S.M. (2018). Recruitment of 7SL RNA to Assembling HIV-1 Virus-like Particles. Traffic.

[173] Strebel K., Khan M.A. (2008). APOBEC3G Encapsidation into HIV-1 Virions: Which RNA Is It?. Retrovirology.

[174] Hell S.W., Wichmann J. (1994). Breaking the Diffraction Resolution Limit by Stimulated Emission: Stimulated-Emission-Depletion Fluorescence Microscopy. Opt. Lett..

[175] Rust M.J., Bates M., Zhuang X. (2006). Sub-Diffraction-Limit Imaging by Stochastic Optical Reconstruction Microscopy (STORM). Nat. Methods.

[176] Betzig E., Patterson G.H., Sougrat R., Lindwasser O.W., Olenych S., Bonifacino J.S., Davidson M.W., Lippincott-Schwartz J., Hess H.F. (2006). Imaging Intracellular Fluorescent Proteins at Nanometer Resolution. Science.

[177] Feng H., Wang X., Xu Z., Zhang X., Gao Y., Gu J., Wang X. (2018). Super-Resolution Fluorescence Microscopy for Single Cell Imaging. Single Cell Biomedicine.

[178] Han R., Li Z., Fan Y., Jiang Y. (2013). Recent Advances in Super-Resolution Fluorescence Imaging and Its Applications in Biology. J. Genet. Genom..

